# L-Cell Expression of Melanocortin-4-Receptor Is Marginal in Most of the Small Intestine in Mice and Humans and Direct Stimulation of Small Intestinal Melanocortin-4-Receptors in Mice and Rats Does Not Affect GLP-1 Secretion

**DOI:** 10.3389/fendo.2021.690387

**Published:** 2021-08-05

**Authors:** Rune E. Kuhre, Ida M. Modvig, Sara L. Jepsen, Hüsün S. Kizilkaya, Cecilie Bæch-Laursen, Christopher A. Smith, Frank Reimann, Fiona M. Gribble, Mette M. Rosenkilde, Jens J. Holst

**Affiliations:** ^1^Department of Biomedical Sciences, Faculty of Health and Medical Sciences, University of Copenhagen, Copenhagen, Denmark; ^2^Novo Nordisk Foundation Center for Basic Metabolic Research, Faculty of Health and Medical Sciences, University of Copenhagen, Copenhagen, Denmark; ^3^Department of Obesity Pharmacology, Novo Nordisk, Måløv, Denmark; ^4^Metabolic Research Laboratories and Medical Research Council Metabolic Diseases Unit, Wellcome Trust-Medical Research Council Institute of Metabolic Science, Addenbrooke’s Hospital, University of Cambridge, Cambridge, United Kingdom

**Keywords:** alpha-MSH, melanocortin, melanocortin-4-receptor, glucagon-like peptide-1 secretion, L-cells

## Abstract

The molecular sensors underlying nutrient-stimulated GLP-1 secretion are currently being investigated. Peripheral administration of melanocortin-4 receptor (MC4R) agonists have been reported to increase GLP-1 plasma concentrations in mice and humans but it is unknown whether this effect results from a direct effect on the GLP-1 secreting L-cells in the intestine, from other effects in the intestine or from extra-intestinal effects. We investigated L-cell expression of MC4R in mouse and human L-cells by reanalyzing publicly available RNA sequencing databases (mouse and human) and by RT-qPCR (mouse), and assessed whether administration of MC4R agonists to a physiologically relevant gut model, isolated perfused mouse and rat small intestine, would stimulate GLP-1 secretion or potentiate glucose-stimulated secretion. L-cell MC4R expression was low in mouse duodenum and hardly detectable in the ileum and MC4R expression was hardly detectable in human L-cells. In isolated perfused mouse and rat intestine, neither intra-luminal nor intra-arterial administration of NDP-alpha-MSH, a potent MC4R agonist, had any effect on GLP-1 secretion (P ≥0.98, n = 5–6) from the upper or lower-half of the small intestine in mice or in the lower half in rats. Furthermore, HS014—an often used MC4R antagonist, which we found to be a partial agonist—did not affect the glucose-induced GLP-1 response in the rat, P = 0.62, n = 6). Studies on transfected COS7-cells confirmed bioactivity of the used compounds and that concentrations employed were well within in the effective range. Our combined data therefore suggest that MC4R-activated GLP-1 secretion in rodents either exclusively occurs in the colon or involves extra-intestinal signaling.

## Introduction

Glucagon-like peptide-1 (GLP-1) is a gastrointestinal hormone that is secreted from intestinal L-cells after meal intake. It acts to inhibit appetite and potentiate glucose-stimulated insulin secretion (1–3). These actions led to the development of GLP-1 based drugs, which have been used for more than a decade for type-2-diabetes treatment and recently also to induce weight loss. In parallel with the development and refinement of GLP-1 based drugs, research has been directed at uncovering the molecular mechanisms responsible for GLP-1 secretion, aiming to create a therapeutically relevant stimulation of endogenous L-cell secretion by pharmacological means (4, 5). Compared to GLP-1 mono-therapy, this approach would be expected to result in improved weight loss since GLP-1 often co-localizes with the anorectic hormones cholecystokinin and peptide-YY (6–9), potentially leading to a synergistic inhibitory effect on appetite during L-cell stimulation. The molecular mechanisms underlying macro-nutrient stimulated GLP-1 secretion have been studied by several laboratories during the last two decades and consensus models for at least some nutrients, e.g. glucose, have been reached (5). However, regulation of secretion also includes potential endocrine, paracrine and neural inputs and several aspects need clarification. It has been suggested that peptide hormones, derived from pro-opiomelanocortin (POMC) may regulate GLP-1 secretion by binding to MC4R expressed by the L-cell. Thus, in one study, MC4R was found to be the second most highly enriched receptor expressed by mouse L-cells (compared to non-L cells from same anatomical site), and the synthetic MC4R agonist, LY2112688 (10), was found to increase plasma GLP-1 and PYY concentrations in mice *in vivo* after intra-peritoneal injection (11). A similar observation was recently made by Panaro et al., who also showed LY2112688-mediated increase in plasma GLP-1 concentrations in mice. Furthermore, they showed that the increase requires *gcg* expression in the ileum and colon (12), pointing towards that these sites of the intestine are responsible for the increased plasma concentrations. Moreover, it has been shown that humans with natural occurring loss-of-function mutations in *MC4R* exhibit reduced plasma PYY levels (marker of L-cell secretion from the distal intestine) in response to an OGTT, and GLP-1 and PYY secretion from human gut specimens (obtained from duodenum, ileum and colon) increased in response to incubation with the MC4R agonists MK-0493, LY2112688, α-MSH and NDP-α-MSH (13).

Importantly, these reported effects of pharmacological MC4R agonism appear to translate to humans as continuous subcutaneous infusion over 72 h of another MC4R agonist, RM-493, increased plasma GLP-1 concentrations by 50–100% (14). However, while these studies are intriguing and show that MC4R agonists may increase plasma GLP-1 concentrations, they do not reveal whether this action results from direct stimulatory actions on MC4Rs expressed on the L-cell or from other indirect actions. Further studies are needed to investigate this and it also needs to be clarified from which source(s) and under which circumstances MC4R agonists are released physiologically and how any daily dynamics in release relate to daily fluctuations in plasma GLP-1 levels. We therefore investigated MC4R expression in mouse and human intestinal L-cells and investigated whether the MC4R ligand, NDP-α-MSH, would stimulate GLP-1 secretion in a physiologically relevant gut model, the isolated perfused mouse and rat small intestine. Furthermore, using the same model, we investigated whether acute application of the described MC4R antagonist, HS014, would inhibit basal GLP-1 and/or glucose-stimulated GLP-1 secretion in the rat. In addition, we characterized the concentration–response relationship between α-MSH and human MCR4 activation, and compared this to published peripheral plasma concentrations of α-MSH in humans to assess whether circulating α-MSH levels are sufficient to directly interact with and activate MC4R on L-cells.

## Materials and Methods

### Ethical Considerations

The isolated perfused rat and mouse small intestinal studies were conducted with permission from the Danish Animal Experiments Inspectorate (2018-15-0201-01397) and our local Institutional Animal Care and Use Committee (Department of Experimental Medicine, Protocol nr. P18-555 and P-20-067). Experiments were performed in accordance with the guidelines of the Danish legislation governing animal experimentation (1987) and the National Institutes of Health (publication number 85-23) and the European Convention for the Protection of Vertebrate Animals used for Experimental and other Scientific Purposes (Council of Europe No. 123, Strasbourg 1985).

### Measurement of cAMP Production Mediated by Activation of Rat and Human MC4R

COS-7 cells were transiently transfected using the calcium-phosphate precipitation method (15) with either human MC4R or rat MC4R. Since MC4R primarily is a Gαs-coupled receptor (16) intra-cellular cAMP production was used as marker of receptor activation. Procedures are described in details elsewhere (17). In brief, the cells were seeded in white 96-well plates at a density of 3.5 ∗ 10^4^ per well one day after the transfection. The following day, the cells were washed twice with HEPES-buffered saline (HBS) and incubated with HBS and 1 mM 3-isobutyl-1-methylxanthine (IBMX) for 30 min at 37°C (95% O_2_, 5% CO_2_). In order to determine agonistic activities, alpha-MSH and NDP-alpha-MSH were added in increasing concentrations and incubation continued for 30 min at 37°C. In order to determine the antagonistic properties of HS014, the cells were pre-incubated for 10 min with increasing concentrations of HS014 followed by addition of fixed concentrations of alpha-MSH or NDP-alpha-MSH, corresponding to 60–80% max activity, and incubated for an additional 20 min (18). After incubation, cells were thoroughly washed and then lysed. Determination of cAMP was done with a HitHunterTM cAMP XS assay (DiscoverX, Herlev, Denmark) according to manufacturer’s instructions.

### Isolated Perfused Mouse and Rat Small Intestine

Male Wistar rats (~250 g) and male C57BL/6JRj mice (~28 g, 11–12 weeks) were purchased from the Janvier Labs (Le Genest-Saint-Isle, France). Mice and rats were housed with *ad libitum* access to standard chow and water following a 12:12 h light:dark cycle and were acclimatized for a least a week before experiments. On the day of experiment, rats and mice were transferred to our perfusion facility and anesthetized with a subcutaneous injection of hypnorm/midazolam (rats) (0.3 ml/100 g body weight, per ml: 0.08 mg fentanyl, 2.5 mg fluanisone, 0.45 mg Methyl Parahydroxybenzoate, 0.05 mg Propyl Parahydroxybenzoate, Midazolam: 1.25 mg, Matrix Pharmaceuticals, Hellerup, Denmark) or with an intraperitoneal injection of Ketamine/Xylazine (0.1 ml/20 g) (90 mg/kg Ketamine, Ketaminol Vet, MSD Animal Health; 10 mg/kg Xylazine, Rompun Vet., Bayer Animal Health) respectively, to block the activity of pain sensing nerves and to induce surgical anesthesia. For distal perfusions, the rat or mouse was placed on a heated operating table (37°C), the abdominal cavity was opened by a mid-line incision and the large intestine was excised after tying off the supplying vasculature. Furthermore, the upper half of the small intestine was removed. In the rat, the lower half of the small intestine (~50 cm) was retained and perfused *in situ* after insertion of a catheter into the upper mesenteric artery, while perfusion effluent was collected through a catheter inserted in the portal vein. The mouse intestine (10–12 cm of the distal part) was perfused by a slightly different pathways (since the upper mesenteric artery is too small to cannulate), perfusing retrogradely through a catheter inserted in the abdominal aorta. To avoid perfusion of other abdominal organs, the spleen and stomach was removed and the kidneys were excluded from the circulation by tying off the renal arteries. For the perfusion of the proximal small mouse intestine the procedure was the same, however the distal part of the intestine was removed and the most proximal part (~11–12 cm) of the small intestine was left untouched. The length of the retained intestine varied little between experiments (coefficient of variation <15%). The perfusion buffer consisted of a Krebs–Ringer bicarbonate buffer supplemented with 0.1% (w/v) BSA (fraction V), 5% (w/v) dextran T-70 [to balance oncotic pressure (Pharmacosmos, Holbaek, Denmark)], and 3.5 mmol/L glucose, and 5 mmol/L pyruvate, fumarate, and glutamate as well as 10 µmol/l 3-isobutyl-1-methylxanthine (IBMX, cat. no. I5879, Sigma Aldrich, Brøndby, Denmark) and 2 ml/L (rat) or 5 ml/L (mouse) Vamin (a mixture of essential and nonessential amino acids; Fresenius Kabi, Bad Homburg, Germany). pH was adjusted to 7.4–7.5. A vascular flow rate of 7.5 ml/min was used for rat perfusions and 2.5 ml/min for mouse perfusions. Luminal stimulations was done at a flow rate of 2.5 ml/min in rats and 0.035 ml/min in mice. Prior to perfusions, the perfusion buffer was gassed with 95% O_2_ and 5% CO_2_. Perfusions were carried out using a UP100 Universal Perfusion System from Hugo Sachs (Harvard Apparatus, March Hugstetten, Germany), which includes heating to 37°C.

### Test Compounds

Nle(4),D-Phe(7)]α-melanocyte-stimulating hormone (NDP-α-MSH) was from Phoenix Pharmaceuticals, Inc. (Cat. no. 043-06, Burlingame, CA, USA). HS014 was from Tocris (Cat. no. 1831, Abingdon, UK). Human α-MSH, D-glucose and bombesin were from Sigma Aldrich (Cat. no. M4135, G8270 and B4272, Brøndby, Denmark).

### MC4R Expression From Mouse and Human L-Cell RNA Sequencing Datasets

All RNA sequencing data were analyzed using R (version 4.0.3). Datasets included in this analysis are listed in the **Table 1** below.

**Table 1:** RNA sequencing data sets used for investigation of L-cell expression of MC4R.

Citation	RNA sequencing	Species	Region	Number of cells/samples
Glass et al. (19), Molecular Metabolism	Single cell	Mouse	Duodenum	259
Roberts et al. (8), Diabetes	Bulk	Mouse	Duodenum	2
			Ileum	3
			Colon	3
Roberts et al. (8), Diabetes	Bulk	Human	Jejunum	11
			Ileum	2
Billing et al. (20), Molecular Metabolism	Single cell	Mouse	Large intestine (colon and rectum)	635
Goldspring et al. (21), Cell Reports	Bulk	Human	Ileum organoids	8

Bulk RNA sequencing datasets were converted into DESeq2 format (1.30.0; Bioconductor). Transcript lengths were obtained from biomaRt (2.42.1; Bioconductor) and imported into the DESeq2 dataset. Fragments per kilobase million (FPKM) values were exported using DESeq2’s in-built function (fpkm). Bulk RNA-seq data presented in heat maps are log2 (average FPKM across samples).

Single-cell datasets were converted into Seurat format (4.0.2; Bioconductor), filtering for only cells with counts for >200 genes, and only genes with counts for >3 cells. Data were normalized to FPKM manually using gene transcript lengths and total reads per sample. For data from Billing et al. (20), all cells were first clustered to obtain five populations as originally published, and data filtered to the two L-cell populations (Insl5 and Nts). Single-cell RNA-seq data presented in heat maps are log2 (average FPKM across cells).

### RT-qPCR Based Assessment of MC4R Expression in Mouse Small Intestine

cDNA was analyzed from previously described mouse villus and crypt L-cell samples in the duodenum (19), and mouse ileal L-cell samples (8), including corresponding negative samples. Subsequent RT-qPCR was carried out as previously described (22), with three samples per region, and three technical replicates per sample. The following probes obtained from Life Technologies (Paisley, UK) were used: *Gpr119*: Mm00731497_s1; *Mc4r*: Mm00457483_s1. Relative expression of each gene of interest was calculated by comparison to expression of the housekeeper *β-actin*. The cycle threshold (CT) per gene was calculated as the mean over technical replicates, and the difference (ΔCT) calculated by subtracting each gene CT from *β-actin* CT. Relative gene expression is represented in figures as 2^ΔCT^.

### Biochemical Measurements

Total GLP-1 concentrations in venous effluents were quantified using an in-house RIA (code: 89390) (23). The assay employs an antibody that exclusively reacts with the amidated C-terminal domain of GLP-1 and thus measures intact GLP-1 (7–36 amide) and the primary metabolite (9–36 amide) with equal potency and it also measures cleaved sequences potentially resulting from mid-site cleavage [e.g. cleaved by neprilysin (24)]. Samples were assayed non-extracted and concentrations were calculated by a four-parameter interpolation to a standard curve containing synthetic GLP-1 7–36 amide (H-6795-GMP, Bachem, Bubendorf, Switzerland) ranging from 5 to 320 pmol/L. I^125^-labeled GLP-1 7–36 amide (a gift from Novo Nordisk A/S, Bagssværd, Denmark) was used as tracer. The standards were prepared in perfusion buffer and run in parallel with the samples. The experimental detection limit was 1 pmol/L and the coefficient of variation was <6% at 20 pmol/L, allowing detection of secretion rates down to 2.5 fmol/min (mouse) and 7.5 fmol/min (rat).

### Data Presentation and Statistical Analysis

GLP-1 total output (fmol/min) was calculated by multiplying perfusion flow (mouse: 2.5 ml/min and rat: 7.5 ml/min) with the GLP-1 concentration in the perfusion effluents (presented in fmol/L). Hormone outputs are presented as means ± SEM. To test for statistical significance of responses, total GLP-1 outputs were calculated by summing up the outputs during the entire period of stimulus administration (e.g. 15 min of NDP-α-MSH administration); these outputs were compared to the total GLP-1 outputs from the preceding basal period which was of the same duration in case of the rat experiments. In the mouse experiments, the length of the initial baseline period was 10 min whereas stimulation periods were 15 min. In this case, outputs are expressed as average outputs within respective periods (fmol/min). Baseline outputs were calculated based on a similar number of samples from the baseline leading up to first period of test compound stimulation constituting the first baseline and the second baseline being derived from the eight samples immediately before stimulus administration and last seven samples during subsequent baseline period (immediately before BBS stimulation). Baseline outputs were calculated in this manner to control for potential baseline drift over the course of the experiment. EC_50_ and IC_50_ values for MC4R activation/inhibition in the *in vitro* studies were calculated from four-parameter logarithmic fitting. Statistical calculations were performed using GraphPad Prism 7 software (La Jolla, CA, USA), employing one-way ANOVA for repeated measurements followed by Tukey multiple comparison test or Student t-test as indicated in the figure legends. P <0.05 was considered significant. Prior to testing, a D’Agostino–Pearson omnibus normality test was performed to confirm Gaussian distribution of the data. Graphs were constructed in GraphPad Prism 7 (La Jolla, CA, USA) and figures were prepared in an Adobe Illustrator (Adobe Systems Incorporated, San Jose, CA, USA).

## Results

### *In Vitro* Determination of Activity for alpha-MSH, NDP-alpha-MSH and HS014 on Rat and Human MC4R

**Figure 1:**
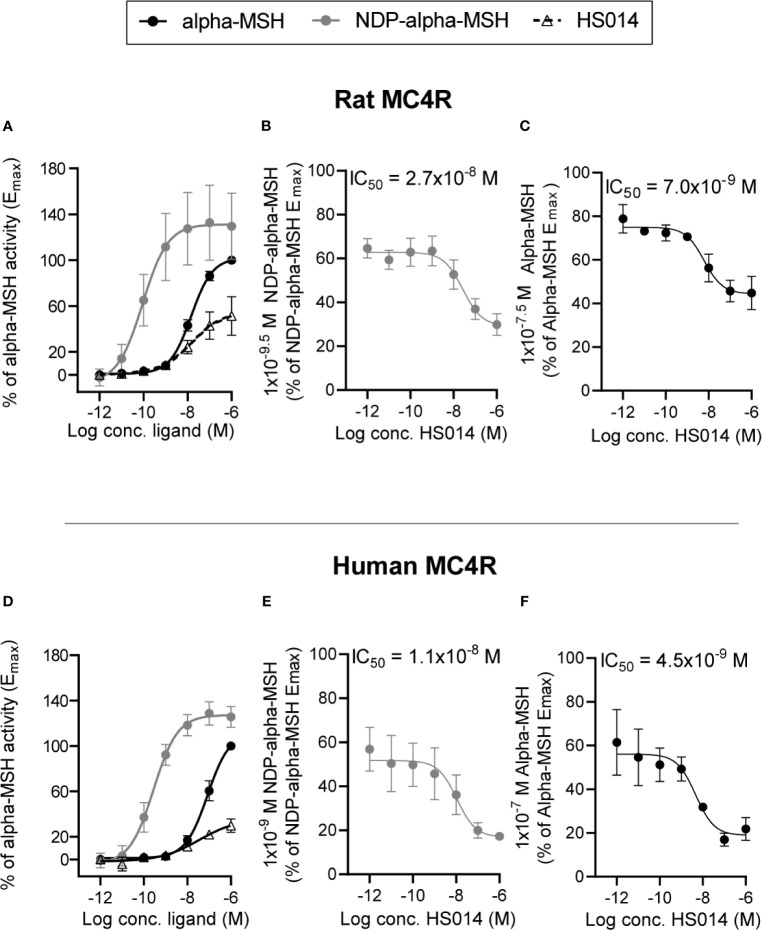
Characterization of agonistic properties of alpha-MSH and NDP-alpha-MSH and antagonizing effects of HS014 on rat and human MC4R in transfected COS-7 cells. Concentration–activation relationship on rat **(A)** and human **(D)** MC4R are shown in response to alpha-MSH (black filled circles with black line), NDP-alpha-MSH (gray circles with gray line) and HS014 (open triangle with staged black line). Concentration-dependent antagonizing effects of HS014 (a partial agonist) on rat **(B, C)** and human **(E, F)** MC4R activation in response to agonist concentrations that result in sub-maximal activation of the receptors. IC_50_-values are indicated above respective graphs. EC_50_ values for **(A)**: alpha-MSH = 1.5 × 10^−8^ M, NDP-alpha-MSH = 9.1^−11^ M, HS014 = 1.5 × 10^−8^ M. EC_50_ values for (×): alpha-MSH = 8.5 × 10^−8^ M, NDP-alpha-MSH = 2.8 × 10^−10^ M, HS014 = 3.5 × 10^−8^ M. Data are presented as means ± SEM, *n* = 3.

In order to use appropriate concentrations of MC4R agonists and antagonist in the experiments on isolated perfused small intestine, we first determined the potencies of alpha-MSH (an endogenous MC4R agonist) and NDP-alpha-MSH (a MC4R super-agonist) in cAMP measurement experiments on rat and human MC4R transfected into COS-7 cells. We moreover determined the inhibitory properties of the MC4R antagonist, HS014 on the two MC4R’s. The rat MC4R was activated by alpha-MSH and NDP-alpha-MSH with EC50 values of 1.5 × 10^−8^ M and 9.1 × 10^−11^ M (LogEC50 −7.8 ± 0.06 and −10.04 ± 0.5, n = 3). NDP-alpha-MSH had an E_max_ that was approximately 120% of that of alpha-MSH (**Figure 1A**, n = 3). HS014 [often described as an MC4R antagonist (25, 26)] was in our setup a partial agonist of rat MC4R with an EC_50_ of 1.5 × 10^−8^ M (LogEC50 −7.8 ± 0.54 here, n = 3) and E_max_ of around 40% of alpha-MSH E_max_. Activation of human MC4R showed similar relationships but with potencies that were slightly lower for all three compounds (alpha-MSH, 8.4 × 10^−8^ log EC50 7.1 ± 0.1, NDP-alpha-MSH, 2.8 × 10^−10^ log EC50 −9.6 ± 0.2, and HS014, 3.5 × 10^−8^ log EC50 7.5 ± 0.8 (**Figure 1D**, *n* = 3). The antagonistic property of HS014 on the rat MC4R was tested using submaximal concentrations of NDP-alpha-MSH or alpha-MSH corresponding to 60–80% of E_max_. Here, an increasing concentration of HS014 revealed inhibition of agonist-induced activity with IC_50_ values of 2.6 × 10^−8^ M and 6.1 × 10^−9^ M (log IC50 7.6 ± 0.3 and −8.2 ± 0.2) (**Figures 1B, C**, *n* = 3). Again, HS014 had comparable effects on human MC4R activity (**Figures 1E, F**, *n* = 3).

### MC4R Expression in Mouse and Human L-Cells

**Figure 2:**
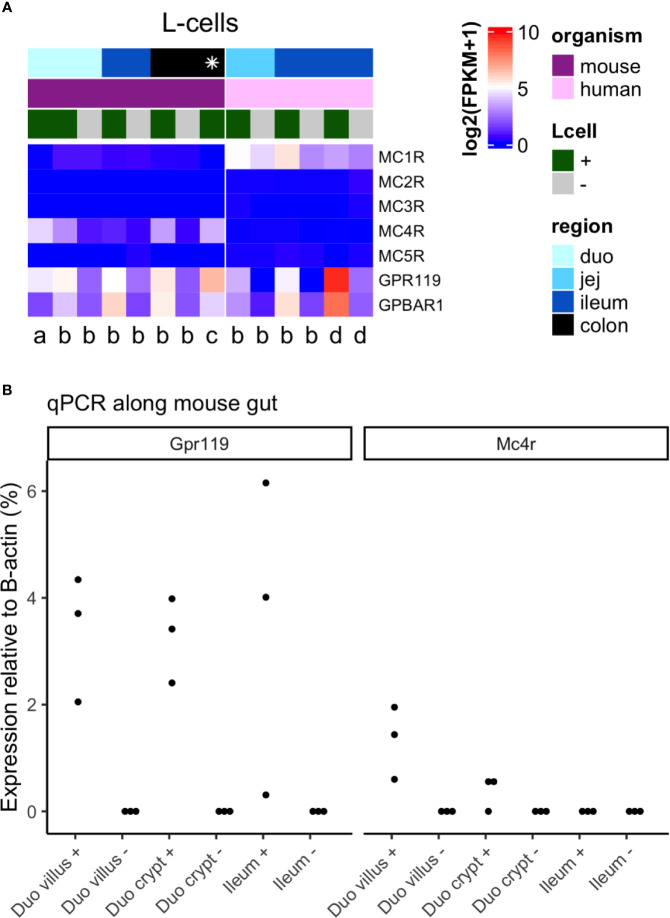
Expression of *Mc4* receptor in mouse and human L-cells. **(A)** Heatmap of melanocortin receptor genes in L-cells from multiple RNA sequencing datasets, including corresponding negative samples where available. Each column is a dataset, the left panel being datasets in mouse, and the right in human. Datasets are labeled per publication [a Glass et al. (19); b Roberts et al. (8); c Billing et al. (20); d Goldspink et al. (21)], per region of the gut (shades of blue, where the distal gut is darker blue; * colon and rectum), and for L-cell positive or negative population (+ = green, − = gray). Data is plotted as log2 (mean fragments per kilobase million, FPKM), increasing from blue → white → red. Data from Billing et al. was calculated as the average across both L-cell clusters (Insl5 and Nts), while data from Glass et al. was calculated as the average across all cells. **(B)** Relative expression of Gpr119 and Mc4r in L-cells from duodenum (duo) villus and crypt, and ileum (n = 3 mice). Samples include both L-cells (+) and their corresponding negatives (−). Values are 2^ΔCT^ relative to β-actin.

To investigate whether any effects of systemic MC4R agonist administration on plasma GLP-1 concentrations in mice (11, 12) and humans (14) may result from direct MC4R-activation at the level of the L-cell, we investigated MC4R expression in mouse and human L-cells by reanalyzing raw data from both bulk RNA sequencing (mouse and human) and single cell RNA sequencing (mouse) (8, 19–21) as well as by qPCR (mouse). In mice, MC4R was expressed at higher levels than the other melanocortin receptor isoforms and, as previously reported, was enriched in L-cells relative to the surrounding epithelium (**Figure 2A**, left panel), which is predominantly constituted from absorptive enterocytes. Expression levels were relatively low and, especially in the large intestine, lower than the expression of other L-cell-expressed GPCRs (GPR119 and bile acid receptor GPBAR1) with established GLP-1 secretory potential. qPCR of mouse duodenum and ileum further demonstrated enrichment of *Mc4r* in duodenal, but not ileal, L-cells, and the low expression relative to *Gpr119* (**Figure 2B**). By contrast, in humans, *MC4R* was barely detectable in the intestinal epithelium, and instead *MC1R* was detectable and enriched in small intestinal L-cells (**Figure 1A**, right panel). Although *MC1R* expression levels were comparable to *GPR119* and *GPBAR1* in acutely isolated human L-cells, in ileal organoid cultures expression levels of *MC1R* were much lower.

### Direct Effects of NDP-alpha-MSH on GLP-1 Secretion From Isolated Perfused Mouse and Rat Small Intestine

As the relatively low *Mc4r* expression, compared with other established pro-secretory GPCRs such as *Gpr119*, puts into question that GLP-1 responses to systemic MC4R agonists observed *in vivo* derive from direct actions on the L-cell, other stimulatory intestinal pathways could drive GLP-1 secretion secondary to intestinal MC4R activation. To assess this possibility, we performed studies on isolated and perfused mouse and rat small intestine.

**Figure 3:**
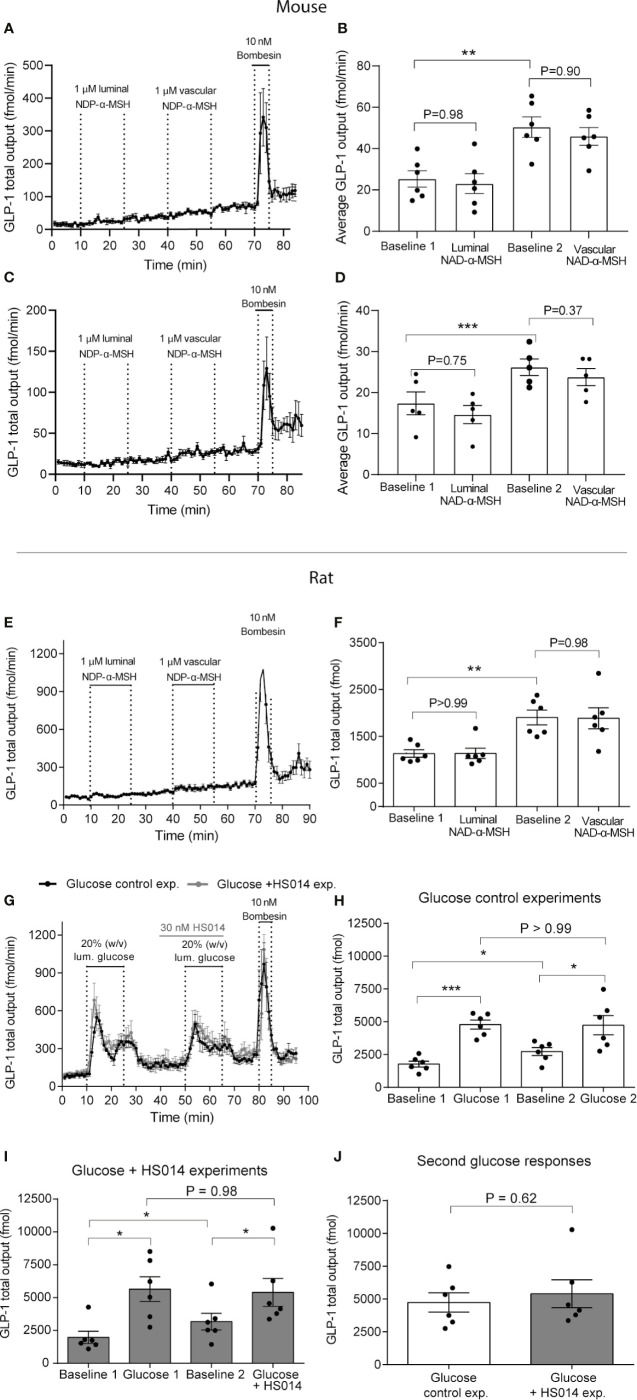
MC4 receptor activity neither controls GLP-1 secretion from isolated perfused mouse and rat small intestine per se nor inhibits glucose-stimulated GLP-1 secretion. GLP-1 (total) outputs are shown in response to luminal or vascular NDP-α-MSH administration in mice **(A–D)** or rats **(E, F)** (1 µM in both cases) or in response to luminal glucose (20%, w/v) with or without co-administration of the MCR-4 partial agonist HS014 (rats, 30 nM) **(G, H)**. Outputs are presented as min–min concentrations (**A–D**, fmol/min) or as total outputs during respective experimental periods (each 15 min of duration) (**E–J**, pmol). Dots in **(B, D, F, H, I, J)** represent outputs from different experiments. Data are shown as means ± SEM. Statistical significance was tested by Student t-test **(H)** or by One-way ANOVA for repeated measurements followed by Tukey *post hoc* test (remaining). P <0.05 was considered significant. *P <0.05, ***P <0.001. n **(A–F)** = 6. Black color in **(G)** are the control glucose experiment, gray line is the glucose + HS014 experiment.

In isolated perfused mouse small intestine—upper half—total GLP-1 secretion during the first baseline period (1–10 min) was on average 25 ± 4.0 fmol/min. Luminal NDP-α-MSH infusion (1 µmol/L) had no effects on GLP-1 output (average secretion: 23 ± 4.8 fmol/min, P = 0.98, n = 6, **Figures 3A, B**). Similarly, vascular NDP-α-MSH administration (1 µmol/L) had no effect on GLP-1 secretion (average outputs; preceding baseline = 50 ± 4.8 fmol/min, during NDP-α-MSH administration = 45.9 ± 4.3 fmol/min, P = 0.90, n = 6, **Figures 3A, B**). Vascular administration of bombesin (positive control) at the end of experiments robustly increased GLP-1 output (**Figures 3C, D**).

Similar secretory dynamics were found when isolating and perfusing the distal part of the mouse small intestine. In this case, average outputs during initial baseline was 17.4 ± 2.4 fmol/min, whereas average output during luminal administration with NDP-α-MSH (1 µmol/L) was 15 ± 2.2 fmol/min, P = 0.75). Consistent with data from the perfused proximal part of the small intestine, vascular administration of NDP-α-MSH at the same dose also did not stimulate GLP-1 output (average output during stimulation: 26 ± 2.0 fmol/min vs. average output during preceding baseline: 24 ± 2.1 fmol/min. P = 0.37). Vascular administration of bombesin (positive control) at the end of experiment, again, robustly increased GLP-1 output (**Figure 3C**).

In isolated perfused rat distal small intestine, total GLP-1 secretion was equally unaffected by luminal and vascular NDP-α-MSH instillation (1 µmol/L; 100-fold higher than the concentration needed to fully activate rat MC4R shown in the *in vitro* studies). In this case, total GLP-1 output was initially (1–15 min) 1.1 ± 0.2 pmol and 1.1 ± 0.1 pmol during subsequent luminal NDP-α-MSH administration (P >0.99, *n* = 6, **Figures 3E, F**). Similarly, vascular (intra-arterial) administration of NDP-α-MSH had no effects on GLP-1 secretion (total output during 15 min of stimulation = 1.9 ± 0.2, total output during immediate preceding baseline period (15 min) = 1.9 ± 0.3 pmol, P = 0.98, **Figures 3E, F**). Baseline secretion drifted slightly upwards during the course of the experiment (P <0.01) in a manner that was unrelated to the administration of test compounds.

### Effects of NDP-alpha-MSH and HS014 on Glucose-Stimulated GLP-1 Secretion From Isolated Perfused Rat Small Intestine

Luminal glucose is a powerful stimulus for GLP-1 secretion. To investigate if the GLP-1 response to glucose is partially driven by MC4R activity, we stimulated the perfused rat small intestine twice with luminal glucose and blocked MC4R activity (by intra-arterial HS014 infusion, 30 nmol/L; >4 fold higher than the IC_50_ for alpha-MSH-stimulated MC4R activation) during the second glucose stimulation (**Figures 3G, H**). In a separate line of control experiments we applied the same protocol but without HS014 infusion. In both experiments, the first glucose stimulation resulted in a robust and immediate increase in GLP-1 secretion which was similar in effect size (total GLP-1 outputs during respective baselines and luminal glucose administration (in both cases 15 min in total) were: control experiment: Baseline = 1.8 ± 0.2 pmol, luminal glucose = 4.8 ± 0.34 pmol, P <0.001, and in the glucose + HS014 experiment: Baseline = 2.0 ± 0.5 pmol, luminal glucose = 5.6 ± 1.0 pmol, P = 0.01, **Figures 3G, H**, *n* = 6). The second glucose response in the control experiment tended to have lower initial peak values compared to the first glucose response, but total secretion during glucose administrations was not different: first response = 4.8 ± 0.3 fmol, second response = 4.7 ± 0.7 fmol, P >0.99, **Figure 3H**). Administration of HS014 together with glucose had no effects on the glucose response. Glucose again robustly increased GLP-1 output and the total output with HS014 was similar to the total output during the first glucose stimulation (total GLP-1 outputs (15 min) were: baseline = 3.2 ± 0.6 pmol, glucose + HS014 = 5.4 ± 1.1 pmol, P <0.05 between baseline and glucose response, P = 0.98 between glucose responses, n = 6, **Figure 3I**). Thus, the total secretory outputs during the second glucose administration were similar between the control experiment and the experiment with co-administration of HS014 (P = 0.62, *n* = 6, **Figure 3J**).

## Discussion

GLP-1 based therapy has proven effective for type-2-diabetes treatment as well as for weight loss. The molecular mechanisms responsible for GLP-1 secretion have been investigated eagerly during the last two decades, and the understanding of the molecular mechanisms underlying macronutrient stimulated GLP-1 secretion has improved (5). Less is known about neural, paracrine and endocrine regulation of GLP-1 secretion. Several stimulators acting *via* G-protein-coupled receptors have been investigated for their ability to stimulate L-cell secretion, including agonists of the bile-acid sensitive receptor GPBAR1 or of the different fatty-acid receptors (22, 27–30). The direct inspiration for the current study came from studies in mice and humans showing that endogenous MC4R ligand, alpha-MSH and/or LY2112688 (an orthosteric MC4R agonist), increased plasma GLP-1 and PYY concentrations in mice after intra-peritoneal injection (11, 12), and that three days of continuous subcutaneous infusion with another MC4R agonist (RM-493) increased plasma GLP-1 concentrations in humans (14). In the mouse study, the underlying mechanism for this response (11) appeared to involve direct activation of L-cell MC4R’s, since MC4R was found to be the second most highly enriched G-protein-coupled receptor expressed by L-cells compared to non-enteroendocrine cells from same intestinal site (11). Data on GLP-1 secretion was, however, not provided, but the authors showed in Ussing chamber experiments on colonic mucosa from mice and humans that alpha-MSH administration to the vascular compartment increased PYY-dependent short circuit currents. The current study was, therefore, designed to investigate whether the reported stimulatory effects of MC4R agonist *in vivo* may result from direct effects on the L-cell, from effects on other gut cells acting within the gut to stimulate GLP-1 secretion, or from extra-intestinal factors. To investigate the structural basis for a direct effect of MC4R activation on GLP-1 secretion, we quantified *Mc4r* expression levels in L-cells from mice and humans, using a collection of publicly available RNA sequencing data bases that include expression levels in L-cells from the mouse duodenum, ileum and colon, and human jejunum and colon, and from non-L-cells from same anatomical sites (a population that is presumably dominated by enterocytes) to assess for potential L-cell enrichment. Furthermore, in the mouse we quantified *Mc4r* expression in isolated L-cells and non-L-cells by RT-qPCR from the duodenum and ileum. The RNA sequencing data showed some L-cell enrichment but relatively low absolute expression of *Mc4r* across the mouse intestine. Expression was detected in a small subset of investigated duodenal cells (39%), similar in size to the subset expressing *Gpr119* (34%), but expression levels of *Mc4r* were 1.25-5 times lower than those of *Gpr119*. This is in direct support of previous findings in mouse proximal small intestine that *Mc4r* is enriched in GLP-1-expressing cells (11), and expressed at levels approximately five times lower than *Gpr119* (11, 31). This lower expression of *Mc4r* compared to *Gpr119* was confirmed by RT-PCR in duodenal L-cells, whereas in ileal L-cells *Mc4r* expression was barely detectable. In human L-cells, *MC4R* expression was similarly hardly detectable and not enriched compared to non-L-cells, whereas we found enriched expression of *MC1R*. Collectively, the expression levels of all MC1-5R’s were in both species either barely detectable or low when compared with other Gs-coupled receptors such as *Gpr119* and *Gpbar1*, which have been shown to robustly stimulate GLP-1 secretion upon activation (22, 28, 31–33).

The regional differences in *Mc4r* expression could suggest that the increased plasma GLP-1 in response to MC4R agonists, if mediated by direct L-cell stimulation, could be restricted to stimulation of L-cells in the very upper part of the small intestine (duodenum). This anatomical site is, however, relative sparse in L-cells and GLP-1 content (34–41), suggesting that this anatomical site contributes minimally to total circulating GLP-1 levels. Furthermore, Panaro et al. (12) have shown that selective knock-out of proglucagon from the distal intestine (ileum and colon, using a Cdx2-Cre x gcg-floxed model) was sufficient to essentially abolish GLP-1 elevation in response to intraperitoneal administration of the MC4R-agonist LY2112688.

As the relative contribution of different intestinal sites in terms of total GLP-1 secretion are not easily assessed *in vivo*, and since secretion *in vivo* may also be regulated by central and neural signaling, it was essential for our functional studies to use an experimental model that would allow study of regional secretion while excluding extra-intestinal signaling but being physiologically relevant. We therefore based our studies on isolated perfused mouse and rat small intestine preparations, which allow studies of GLP-1 secretion at high time resolution and with almost complete experimental control. At the same time, these models maintain epithelial polarization and vasculature as well as local enteric innervation and paracrine interrelationships (42). Despite detectable *Mc4r* expression in upper and, if somewhat lower expression, in lower small intestinal L-cells, we were, however, unable to detect any L-cell stimulation by the MC4R super-agonist NDP-alpha-MSH from either upper or lower small intestinal preparations. This suggests that the previously reported *in vivo* observations either cannot be simulated in this preparation, arise solely from stimulation of MC4R on colonic L-cells or involve MC4R located outside the intestinal preparation. GLP-1 secretion can readily be stimulated from perfused small intestinal preparations by e.g. GPBAR1 agonists (22, 28), arguing that in principle Gs-coupled receptor stimulated GLP-1 secretion is observable in this preparation. It is possible that the *Mc4r* expression, being somewhat lower than *Gpr119* and *Gpbar1* expression, is insufficient to elicit a secretory response. Given that colonic L-cell *Mc4r* expression is at similar relatively low levels as observed in duodenal L-cells it seems thus unlikely that direct stimulation of colonic L-cells alone is underlying *in vivo* GLP-1 responses to MC4R-agonists, although GLP-1 and PYY secretion from *ex vivo* incubated human colonic mucosal specimens was reported to increase in response to MC4R agonists (13). Modulation by extra-intestinal MC4R not present in the preparation is a possibility that should be addressed in future research, especially, given the apparent absence of *MC4R* expression in human L-cells.

While our combined data therefore suggested that intestinal MC4R activation does not in itself drive GLP-1 secretion in the mouse and rat small intestine, MC4R signaling in the gut could potentially still potentiate GLP-1 secretion stimulated *via* other pathways. We therefore next investigated if the GLP-1 response to one of the most powerful and well characterized stimuli—luminal glucose—was affected by simultaneous activation/partial inhibition of MC4R activity. For these studies, we isolated and perfused the distal part of the rat small intestine and stimulated the perfused preparation with luminal glucose with or without simultaneous intra-arterial NDP-alpha-MSH or HS014 infusion. The latter is a frequently described MC4R antagonist but, as we show here in our *in vitro* studies, actually acts as a partial agonist at certain concentrations. However, at the concentration used in our perfusion experiments (30 nM), HS014 acts functionally as an antagonist. Consistent with some of our previous studies, luminal glucose infusion increased GLP-1 secretion from isolated perfused rat small intestine 2–3 fold (43, 44), but neither NDP-alpha-MSH (at 100-fold higher than E_max_) nor HS014 affected the response, despite the concentration of the latter being, based on our pharmacology studies, well above the dose expected to be required [two to three times higher than the IC_50_ of alpha-MSH on rat MC4R, and corresponding to the reported IC_50_ on human MC4R (3–4 nM) (45)]. Although, frequently reported to be a specific MC4R antagonist, HS014 also antagonizes MC1R, MC3R and MC5R but with IC50’s that for the human receptor-subtypes are 15–200 fold higher than the IC_50_ for MC4R (~55 nM) (45).

## Concluding Remarks

The expression data and perfusion data we report here do not support that MC4R-activation in the small intestine or at the level of the small intestinal L-cells should affect GLP-1 secretion. Nevertheless, an important question that remains to be clarified with regards to the potential importance of gut MC4R signaling for GLP-1 secretion is the potential source of melanocortin receptor ligands in the intestine. Peptides of intestinal bacterial origin with melanocortin receptor activity have been reported (46), but these are unlikely to reach high concentrations in the small intestine, where we found the highest *Mc4r* expression in the mouse. It is questionable whether plasma alpha-MSH would activate L-cell melanocortin receptors in humans since we found the EC_50_ for alpha-MSH on human MC4R to be 85 nM, while the plasma concentrations of alpha-MSH in humans are more than 5,000 fold lower (~4–15 pM (47–49). Others have found the EC_50_ of α-MSH on human MC4R to be even higher; ~900 nM (45). POMC positive cells have, however, been reported in the human intestine and although alpha-MSH and ACTH were not detected, beta-MSH was (35, 50), suggesting that a local intestinal source of MC4R agonist may exist. The functional role of such a potential source and the control regulating its secretion warrants further investigation.

## Data Availability Statement

RNA sequencing raw data are available through the indicated sources. Other raw data can be sent upon reasonable request to the corresponding authors.

## Ethics Statement

The animal study was reviewed and approved by The Danish Animal Experiments Inspectorate (2018-15-0201-01397).

## Author Contributions

RK, IM, and JH conceived the study. HK and MR did all work related to the pharmacological studies on rat and human MC4R. RK, SJ, CB-L, and IM designed and performed isolated perfused small intestine perfusions. RK, IM, SJ, CB-L, and JH interpreted data. CS, FR, and FG did all work related to the expression data. RK drafted the manuscript. IM, SJ, CB-L, HK, CS, FR, FG, MR, and JH critically revised it and provided substantial intellectual content. All authors contributed to the article and approved the submitted version.

## Funding

The study was supported by postdoctoral scholarships to RK from Lundbeck foundation (Lundbeckfonden, R264-2017-3492) and from Weimann Foundation (Købmand i Odense Johann og Hanne Weimann født Seed fonden), a running cost grant from Lundbeck foundation to RK (Lundbeckfonden, R289-2018-1026), an unrestricted grant to JH from the Novo Nordisk Center for Basic Metabolic Research (Novo Nordisk Foundation, Denmark) and from another Novo Nordisk grant to JH (no. NNF15OC0016574) as well as an additional grant to JH from the European Research Council under the European Union’s Horizon 2020 research and innovation program (grant agreement no. 695069-BYPASSWITHOUTSURGERY). SJ was supported by a postdoctoral grant from Independent Research Fund Denmark (Danmarks Frie Forskningsfond) (grant no. 0129-00002B). Work in the laboratory of FR and FG was supported by the Wellcome Trust (106262/Z/14/Z, 106263/Z/14/Z) and the UK Medical Research Council (MRC_MC_UU_12012/3). The studies done in the laboratory of MR was supported by donation from deceased Valter Alex Torbjørn Eichmuller (VAT Eichmuller—2020-117043) and Kirsten and Freddy Johansen’s Foundation (KFJ-2017-112697).

## Conflict of Interest

RK is employed by Novo Nordisk (Denmark), but was exclusively employed at University of Copenhagen during the conception and design of experiments forming the basis of this paper. Novo Nordisk was not involved in the conception of study, design and execution of the experiments, interpretation of data or writing of the manuscript.

The remaining authors declare that the research was conducted in the absence of any commercial or financial relationships that could be construed as a potential conflict of interest.

## Publisher’s Note

All claims expressed in this article are solely those of the authors and do not necessarily represent those of their affiliated organizations, or those of the publisher, the editors and the reviewers. Any product that may be evaluated in this article, or claim that may be made by its manufacturer, is not guaranteed or endorsed by the publisher.
